# Supplementary material for: Polymeric Delivery Systems as a Potential Vaccine against Visceral Leishmaniasis: Formulation Development and Immunogenicity
Source: Vaccines (Basel). 2023 Jul 31;11(8):1309. doi: 10.3390/vaccines11081309 (PMC10459565; doi:10.3390/vaccines11081309)
Supplement: Supplementary file 1 [file vaccines-11-01309-s001.zip › vaccines-2394185-supplementary.pdf]

Supplementary Data 1

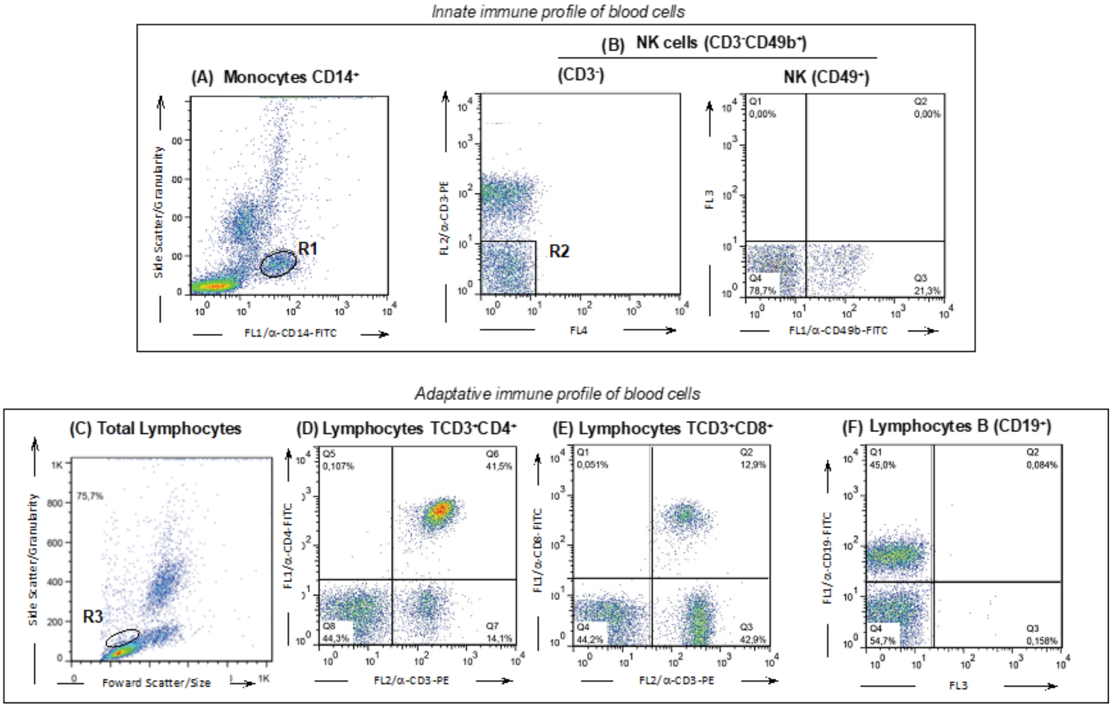

Figure S1. Innate and adaptative immune profile of blood cells was performed by flow cytometry. Phenotypic studies were performed using anti-CD14-FITC mAbs to identify (A) monocytes (Site scatter $CD14^{+}$ - gate R1); anti-$CD3^{-}CD49b^{+}$ monoclonal antibodies (mAbs) to (B) NK–cells ($CD3^{-}$ lymphocytes- gate R2) and posteriorly anti-$CD49b^{+}$-FITC. Total lymphocytes (C) were evaluated by R3 gate constructed from the graph of size *versus* granularity and from this R3 gate were evaluate T-cells subsets - (D) $TCD3^{+}CD4^{+}$ (anti-$CD3^{+}$PE/anti$CD4^{+}$FITC mAbs) and (E) $TCD3^{+}CD8^{+}$ (anti-$CD3^{+}$PE/anti-$CD8^{+}$FITC mAbs) lymphocytes. Finally, a graph was made to evaluate the population of (F) B lymphocytes (anti-$CD19^{+}$FITC mAbs).

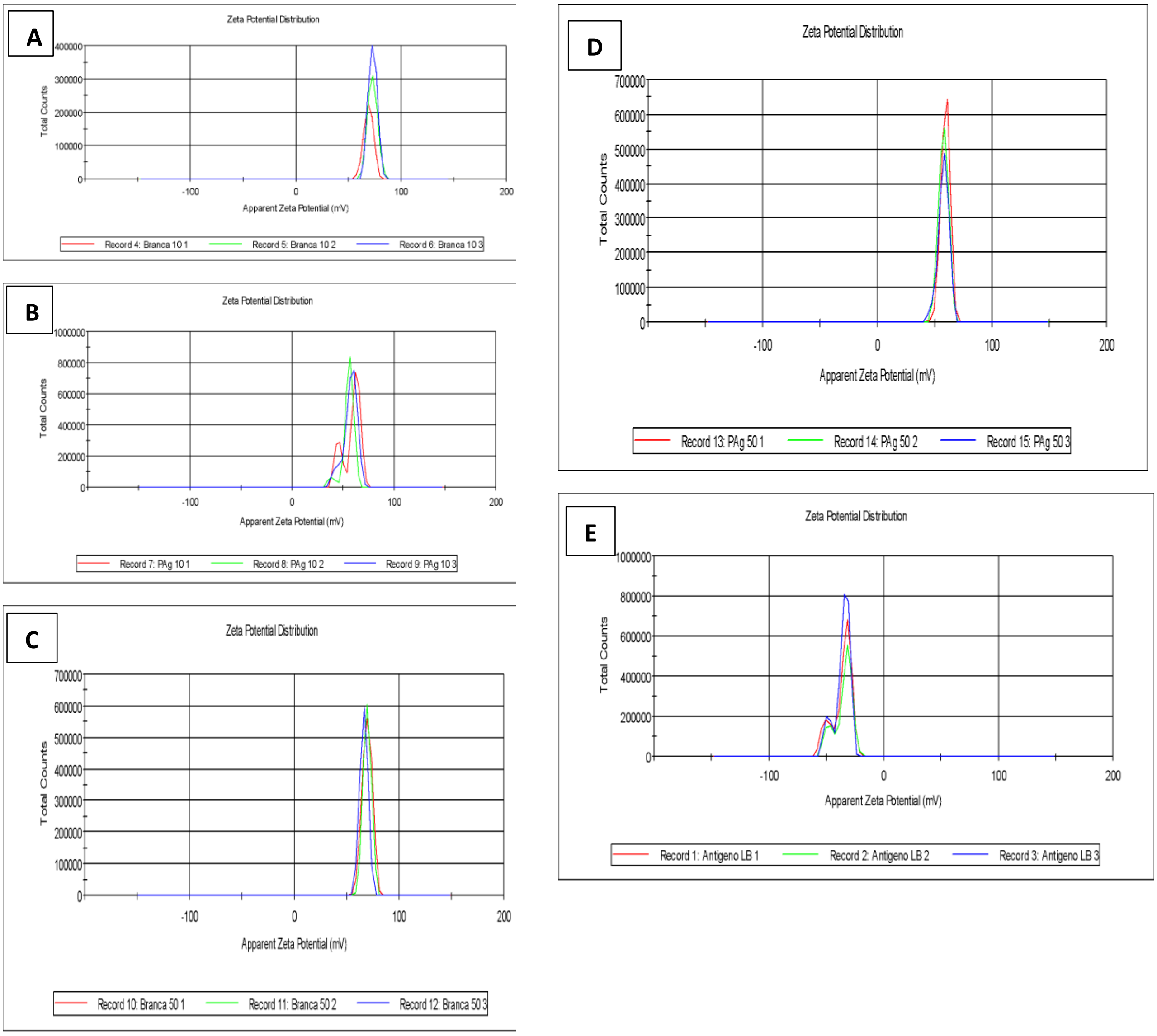

Figure S2. Zeta potential measurements of the different formulations of particles. (A) Blank SMP1: mean +71.6 mV, SD 2.23 mV; **(B)** Ag-loaded SMP[1]:mean +57 mV, SD 1.46 mV, **(C)** Blank SMP2:mean +68.4 mV, SD 1.7 mV; **(D)** Ag-loaded SMP2: mean +58.1 mV, SD 1.01 mV; **(E)** Antigen Protein aggregates: mean -35.7mV, SD 0.643.
